# Optimization of the Preparation Process of Crosslinked Polyvinyl Alcohol and Its Thermal Stability in Cementing Slurry

**DOI:** 10.3390/gels11020098

**Published:** 2025-01-30

**Authors:** Junhao Li, Haochen Ai, Qingchen Wang, Huifeng He, Xiaofeng Chang, Gang Chen, Alena Golian-Struhárová, Michal Slaný, Fangling Qin

**Affiliations:** 1 Engineering Research Center of Oil and Gas Field Chemistry, Universities of Shaanxi Provence, Xi’an Shiyou University, Xi’an 710065, Chinagangchen@xsyu.edu.cn (G.C.); 2 Shaanxi Province Key Laboratory of Environmental Pollution Control and Reservoir Protection Technology of Oilfields, Xi’an Shiyou University, Xi’an 710065, China; 3 Xi’an Origin Chemical Technologies Co., Ltd., Xi’an 710061, China; 4 Changqing Drilling Company of CCDC, China National Petroleum Corporation, Xi’an 710060, China; 5 Department of Materials Engineering and Physics, Faculty of Civil Engineering, Slovak University of Technology, Radlinského 11, 810 05 Bratislava, Slovakia; 6 Department of Materials Engineering and Chemistry, Faculty of Civil Engineering, Czech Technical University in Prague, Thákurova 7, 166 29 Prague, Czech Republic

**Keywords:** polyvinyl alcohol, crosslinked, oil well cement slurry, fluid loss additive

## Abstract

This study focuses on addressing the limitations of fluid loss additive in cement slurry under higher temperatures. The synthesis process of glutaraldehyde-crosslinked polyvinyl alcohol (PVA) was optimized to develop an efficient fluid loss additive for oil well cement slurries. Using one-factor experiments and the uniform design method, the optimal synthesis parameters were established: a reaction temperature of 50 °C; an acid concentration of 1 mol/L; a PVA mass concentration of 8%; a molar ratio of glutaraldehyde to PVA hydroxyl group of 1.47; and a crosslinking degree of 1.49%. The optimized crosslinked PVA demonstrated the ability to control API fluid loss within 50 mL when applied at 1% concentration in cement slurry under conditions of 30–110 °C and 6.9 MPa. Rheological analysis at medium and high temperatures revealed improved slurry properties, including smooth thickening curves and unaffected compressive strength. Further analyses, including thermogravimetric analysis (TGA), Zeta potential testing, and scanning electron microscopy (SEM), revealed that the crosslinked PVA hydrogel remained thermally stable up to 260 °C. Chemical crosslinking transformed the linear PVA into a network structure, enhancing its molecular weight, viscoelasticity, and thermal stability. This thermal resistance mechanism is attributed to the hydrogel’s high-strength reticular structure which forms a uniform, dense, and highly stable adsorption layer, thereby improving both the additive’s efficiency and the hydrogel’s temperature resistance.

## 1. Introduction

As the development of oil and gas wells advances toward greater depths and ultra-deep formations, the demands placed on additives for oil well cement are significantly increasing. During the cementing process, the free water in cement slurry often escapes in substantial quantities to the surrounding formation due to high temperatures. This makes controlling fluid loss in cement slurry a critical factor, as it directly impacts both cementing quality and operational costs. Fluid loss can be effectively mitigated by incorporating fluid loss additives, which ensure proper zonal isolation and cementing quality.

Generally speaking, the mechanism of action of fluid loss additives can generally be attributed to three primary aspects [1,2]: First, fluid loss additives form a physical barrier. These additives adsorb onto the surfaces of cement particles or aggregates, forming a barrier or depositing within pores, which restricts fluid escape. Second, they alter the charge state of cement particles. By interacting with cement particle surfaces, the additives modify their charge state, thereby reducing water exchange between the cement slurry and the surrounding medium. Third, they form a thin film on the cement particle surfaces, which prevents water from escaping rapidly into the formation.

Common fluid loss additives include particulate materials, cellulose derivatives, and synthetic polymers [3]. Environmentally friendly options, such as cellulose derivatives, are suitable for well operation at medium to low temperatures (40–90 °C). However, these natural polymer materials and their derivatives are generally limited in their application to wells within this temperature range [4]. Acrylamide copolymers present challenges in separating the extracted liquid, and their poor degradability restricts their application [5].

PVA is widely used as a fluid loss additive in cementing slurries [6]. It is a water-soluble polymer that forms a hydrogel within the cement slurry, functioning as an effective fluid loss additive [7,8] and as a reinforcement agent for cement structures [9]. PVA is rich in hydroxyl groups, which allow it to adsorb onto the surface of cement particles, forming an impermeable, compact filter cake during filtration. It significantly reduces fluid loss. However, when used alone, the hydrogen bonds formed by its hydroxyl groups within and between molecules are prone to disruption at high temperatures [10,11]. Consequently, PVA struggles to form a stable film between the filter cake and the surrounding medium, leading to reduced efficiency as a fluid loss additive. Thus, its application is typically limited to formations at temperatures below 80 °C. Additionally, the large amount of PVA required for effective fluid loss control renders its use economically unfeasible.

To address these challenges, research has focused on chemically modifying PVA to enhance its temperature resistance [12,13,14]. Building on prior studies, this study optimized the synthesis process of crosslinked PVA and evaluated its efficiency as a fluid loss additive in high-temperature cement slurries. This study also assessed the rheological properties, thickening behavior, and compressive strength of the cement slurry after solidification. Furthermore, crosslinked PVA was characterized using thermogravimetric analysis, scanning electron microscopy, and other methods. Finally, the fluid loss additive mechanism of the crosslinked PVA hydrogel was systematically investigated.

## 2. Results and Discussion

### 2.1. Optimization of the Synthesis Process of Crosslinked PVA

The initial step involved the application of the single-factor method to evaluate the influence of individual process conditions on the crosslinking reaction of PVA. However, this method only considers the impact of one factor at a time and may not comprehensively elucidate which factor has a more significant effect on the crosslinking degree of PVA. Drawing from the on-site construction context of the Drilling and Production Engineering Technology Research Institute of Chuanqing Drilling Engineering Co., Ltd., the reaction temperature, acid concentration, PVA concentration, and molar ratio of glutaraldehyde to PVA hydroxyl groups were identified as the key variables for investigation. The subsequent step was to determine the primary influencing factors using the uniform design method, which not only reduces the experimental frequency but also ensures the accuracy of the experimental results.

#### 2.1.1. Influence of Reaction Temperature on Crosslinking Degree

**Figure 1:**
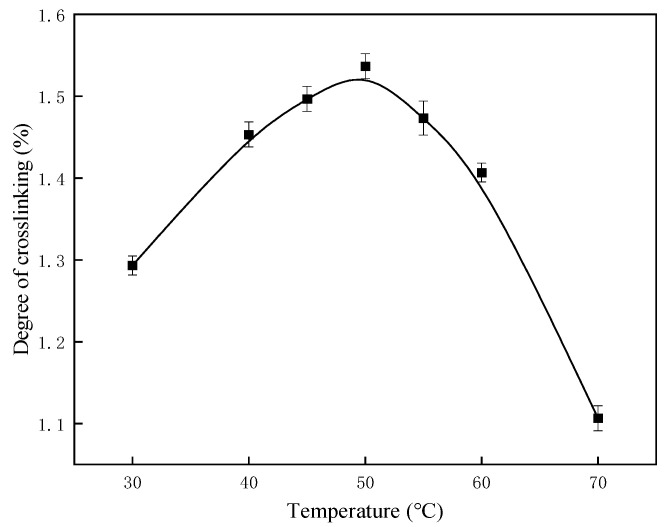
Effect of reaction temperature on crosslinking degree.

Using a PVA mass fraction of 10%, an acid concentration of 1 mol/L, and a molar ratio of 1.55 between glutaraldehyde and PVA hydroxyl groups, the effect of reaction temperature on the crosslinking degree was investigated. The results, presented in Figure 1, demonstrate that the crosslinking degree initially increases with rising temperature but decreases after reaching an optimum value at 50 °C. This temperature corresponds to the highest observed crosslinking degree.

As the polymerization reaction progresses, molecular structures transition from a disordered, free state to a more ordered configuration, resulting in a decrease in overall entropy. At lower temperatures, the Gibbs free energy change (ΔG) of the polymerization reaction becomes more negative, favoring a spontaneous reaction. Therefore, reaction temperature is a critical factor in determining the efficiency of polymer synthesis.

#### 2.1.2. Impact of Acid Concentration on Crosslinking Kinetics and Final Crosslinking Degree

**Figure 2:**
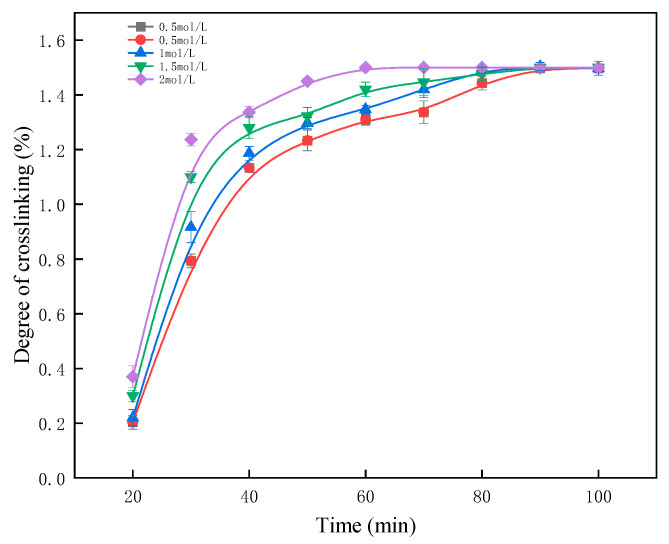
Effect of reaction time on crosslinking degree under different acid concentrations.

Under conditions of a 10% PVA mass fraction, a reaction temperature of 50 °C, and a molar ratio of 1.55 between glutaraldehyde and PVA hydroxyl groups, the influence of acid concentration on the crosslinking reaction was analyzed. As shown in Figure 2, the crosslinking degree increases continuously with reaction time, eventually reaching a plateau at its maximum value. Notably, as acid concentration increases, the reaction reaches this maximum crosslinking degree more rapidly.

While a higher acid concentration accelerates the reaction kinetics, it has no discernible impact on the final crosslinking degree of the synthesized crosslinked PVA gel. Thus, acid concentration primarily affects the rate of polymerization without altering equilibrium crosslinking degree.

#### 2.1.3. Influence of Monomer Mass Fraction on Crosslinking Degree

**Figure 3:**
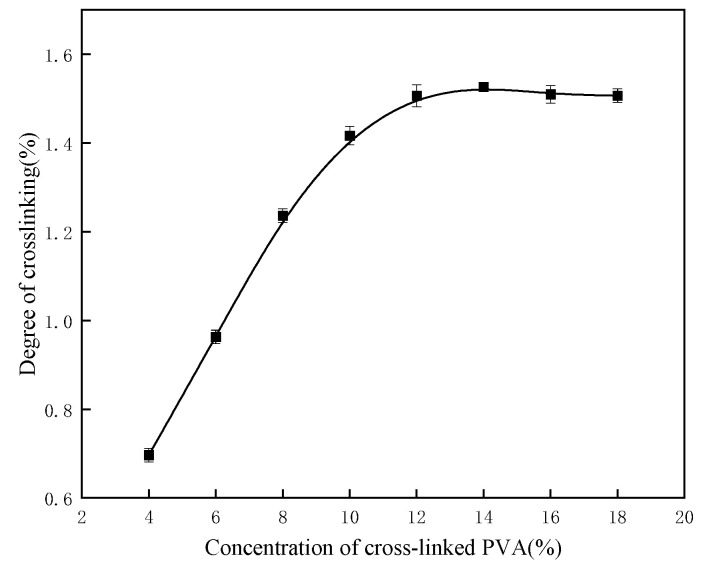
Influence of PVA mass fraction on crosslinking degree.

The effect of monomer mass fraction on the crosslinking degree was studied at a reaction temperature of 50 °C, an acid concentration of 1 mol/L, and a molar ratio of glutaraldehyde to PVA hydroxyl groups of 1.55. Figure 3 illustrates that as the monomer mass fraction increases, the crosslinking degree initially rises, reaching a peak value at a PVA mass fraction of 14%. At this concentration, the crosslinking degree achieves its maximum value of 1.53.

#### 2.1.4. Effect of Molar Ratio of Glutaraldehyde to PVA Hydroxyl Groups on Crosslinking Degree

**Figure 4:**
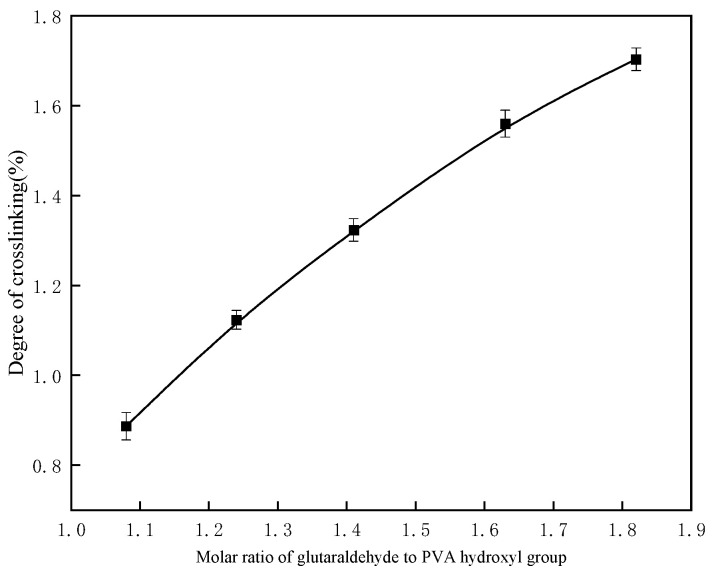
Effect of molar ratio of glutaraldehyde to PVA hydroxyl groups on crosslinking degree.

Using a reaction temperature of 50 °C, an acid concentration of 1 mol/L, and a PVA mass fraction of 10%, the influence of the ratio of glutaraldehyde to PVA hydroxyl groups on the crosslinking degree was evaluated. Figure 4 shows a clear upward trend in the crosslinking degree with an increasing proportion of glutaraldehyde. This behavior is attributed to chemical crosslinking, which converts the linear PVA structure into a three-dimensional network.

As the glutaraldehyde content increases, more hydroxyl groups are engaged in the crosslinking reaction, creating additional crosslinking points. These points bind the PVA chains together, restricting their degrees of freedom and enhancing the rigidity and stability of the network structure. This increase in crosslinking points strengthens the three-dimensional network, improving the mechanical integrity and reducing the swelling capacity of the polymer [15].

However, further increasing the molar ratio of glutaraldehyde to PVA hydroxyl groups leads to excessive crosslinking and the phenomenon known as “burst polymerization”. This phenomenon arises due to two potential reasons: One possibility is that a higher glutaraldehyde concentration accelerates the reaction rate, causing localized areas of intense reaction, which can result in a “burst” [16]. Another possibility is that at elevated molar ratios, the rapid formation of a gel phase at a critical point may trap unreacted monomers and crosslinking agents within the gel. This entrapment leads to continued reaction within the gel, producing a larger polymer network and ultimately causing “burst polymerization” [17].

To avoid such issues, it is essential to carefully control the molar ratio of glutaraldehyde to PVA hydroxyl groups during polymer gel synthesis. This ensures the formation of a stable network structure without triggering “burst polymerization”.

#### 2.1.5. Optimization of Reactions Using the Uniform Design Method

To evaluate the crosslinking degree, a uniform design approach was employed to optimize the synthesis conditions for the PVA fluid loss additive. The design scheme and optimal synthesis parameters are detailed in Table 1 and Table 2. Based on the factors affecting the crosslinking degree, reaction temperature (Factor A), PVA mass fraction (Factor B), and the molar ratio of glutaraldehyde to PVA hydroxyl groups (Factor C) were selected as the primary variables for investigation.

A U_7_(7^4^) uniform design was implemented to systematically analyze these factors. The experimental data were processed to derive a regression equation: y = −1.77 + 0.00031X_A_^2^ + 0.211X_B_ X_C_. The correlation coefficient R= 0.9958, the test value F = 69.66, and the significance level α = 0.05 demonstrate a high level of consistency in the data. The regression equation reveals that reaction temperature exerts a relatively minor influence on the polymer’s crosslinking degree. In contrast, the PVA mass fraction and the molar ratio of glutaraldehyde to PVA hydroxyl groups exhibit a significant and synergistic effect on crosslinking performance.

### 2.2. Temperature Resistance

**Figure 5:**
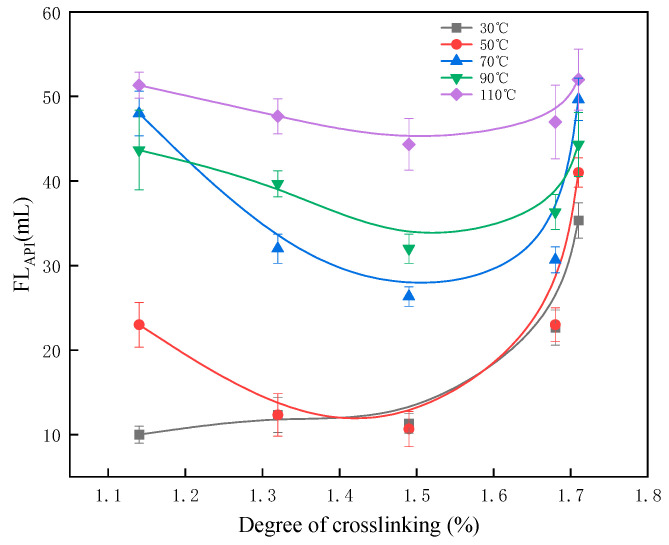
The influence of the crosslinking degree of crosslinked PVA on filtration loss.

The effect of temperature on the fluid loss performance of PVA with varying crosslinking degrees was further evaluated to determine the optimal crosslinking degree. The cement slurry formulation used in the experiment consisted of Jiahua G-grade oil well cement + 1% crosslinked PVA, with a tap water-to-cement ratio of 44%. The experiments were conducted at temperatures of 30, 50, 70, 90, and 110 °C under a pressure of 6.9 MPa. The experimental results are presented in Figure 5.

The results indicate that fluid loss initially decreases and then increases as the crosslinking degree rises, with minimum fluid loss observed at a crosslinking degree of 1.49% across all tested temperatures. This trend can be attributed to the denser network structure formed as the crosslinking degree increases, which enhances fluid loss performance. However, when the crosslinking degree exceeds the optimal value, fluid loss increases due to over-crosslinking. It reduces the ability of the polymer gel to disperse or dissolve effectively in the cement slurry, impairing its pore-blocking capability.

Furthermore, fluid loss of the PVA additive increases with rising temperatures. Higher temperatures reduce the viscosity of the crosslinked PVA gel, weakening its ability to retain free water and block voids in the cement particles. Despite this, fluid loss across all tested conditions was maintained at or below 50 mL, meeting the requirements of on-site cementing applications.

### 2.3. Effect of Crosslinked PVA Concentration on Water Loss

**Figure 6:**
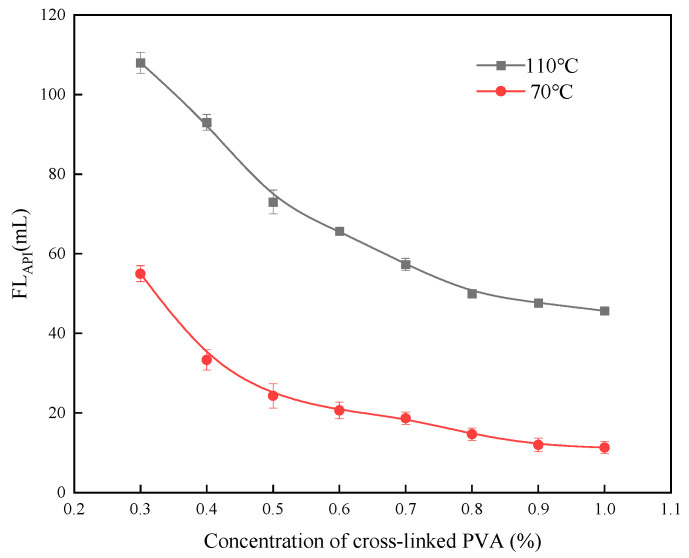
The effect of crosslinked PVA concentration on fluid loss performance.

The influence of the concentration of crosslinked polyvinyl alcohol (PVA) on fluid loss performance at medium and high temperatures was investigated. The cement slurry formulation used was Jiahua G-grade oil well cement, with varying percentages of crosslinked PVA and tap water, and a constant 44% water-to-cement ratio. The experimental results are shown in Figure 6.

At medium and high temperatures, fluid loss in the cement slurry decreases as the concentration of crosslinked PVA hydrogel increases. At 70 °C, a PVA concentration of 0.4% is sufficient to achieve fluid loss values of ≤50 mL. At 110 °C, a PVA concentration exceeding 0.8% meets construction standards, indicating that the hydrogel possesses excellent temperature resistance when incorporated into the cement slurry.

### 2.4. Rheological Properties

The fluid loss additive effect of crosslinked PVA on cement slurry primarily operates through hydrogel binding with free water and the plugging of voids between cement particles [18]. While increasing the viscosity of the hydrogel component in cement slurry can effectively reduce fluid evaporation, it may also negatively affect the slurry’s flowability. Consequently, evaluating the rheological behavior of cement slurry after incorporating crosslinked PVA is crucial.

Cement slurry behaves as a non-Newtonian fluid, and its rheological characteristics are described using the Flow Behavior Index (n) and Consistency Index (K).The governing equations are outlined below (Equations (1) and (2)). The influence of varying concentrations of crosslinked PVA on the Flow Behavior Index (n) and Consistency Index (K) of cement slurry was assessed, as shown in Table 3.

Under curing conditions of 50 °C, 80 °C, and 110 °C, the addition of crosslinked PVA increases the Flow Behavior Index (n) while decreasing the Consistency Index (K). These changes indicate enhanced flowability and reduced viscosity of the cement slurry, which are advantageous for construction applications, particularly at cementing sites.

This improvement is attributed to the hydroxyl-rich nature of crosslinked PVA, which facilitates adsorption on the surface of cement particles, creating a tight encapsulation layer around them [19]. This adsorption process enhances particle dispersion, prevents aggregation and crosslinking, and subsequently improves slurry flowability.

As the concentration of the fluid loss additive increases, the changes in slurry flowability are relatively minor, but the consistency coefficient (g) gradually rises. This phenomenon can be attributed to the increased content of crosslinked PVA, wherein the hydrogel contributes to a rise in slurry viscosity.

Additionally, with higher curing temperatures, the improvement in flowability and the reduced viscosity of the cement slurry are likely due to the temperature-induced reduction in the viscosity of the crosslinked PVA hydrogel. This decrease weakens its ability to bind free water effectively, thereby reducing the overall viscosity of the slurry [20].

(1) $$n=2.092\mathrm{lg}\left(\frac{\theta_{600}}{\theta_{300}}\right)$$

(2) $$K=0.511\frac{\theta_{300}}{511^{n}}$$

where n represents the Flow Behavior Index (a higher value of n indicates greater fluidity of the cement slurry) and K is the Consistency Index (a higher K value signifies a thicker cement slurry). θ_600_ represents the viscosity at 600 rpm, while θ_300_ represents the viscosity at 300 rpm.

### 2.5. Compressive Strength Test

Compressive strength is a critical parameter representing the maximum pressure that cement can withstand per unit area. It serves as a key indicator of the performance and durability of well cementing materials. The inclusion of additives in cement slurry can influence the compressive strength of hardened cement; hence, a thorough evaluation is essential.

The basic formulation for the cement slurry includes Jiahua G-grade oil well cement + 1% crosslinked PVA + tap water (maintaining a 44% water-to-cement ratio). The slurry samples were cured for 24 h at different temperatures, and compressive strength was measured, as detailed in Table 4.

The results indicate that incorporating crosslinked PVA significantly enhances the compressive strength of hardened cement. Additionally, the compressive strength improves as the curing temperature increases. These findings suggest that crosslinked PVA not only boosts the mechanical integrity of cement but also ensures high performance at elevated temperatures.

The compressive strength values at all tested temperature ranges meet the on-site construction requirement of ≥14 MPa, demonstrating the multifunctional advantages of crosslinked PVA in cementing applications.

### 2.6. Stability of Cement Slurry

During the extended waiting period for solidification in the cementing process, the significant density difference between cement particles and free water can lead to particle separation, resulting in settling and layered deposition [21,22,23]. When cement particles precipitate, the density of the oil well cement slurry varies at different depths in the well, negatively impacting the strength development and thickening time of the slurry, thereby affecting the quality of well cementing.

The settling stability of the cement slurry is evaluated by measuring the amount of free water released. This study assesses the effect of crosslinked PVA on water release at medium and high temperatures. The cement slurry formulation used is Jiahua G-grade oil well cement + x% crosslinked PVA + water (water-to-cement ratio of 0.44). The test results are presented in Table 5.

The addition of crosslinked PVA significantly reduces the free liquid content in the cement slurry. As the crosslinked PVA concentration increases, the amount of free liquid decreases, reaching zero at all curing temperatures when the concentration reaches 1.5%. This improvement can be attributed to the hydrogel network structure formed by crosslinked PVA, which effectively binds free water and prevents its precipitation.

### 2.7. Thickening Performance

The thickening rate of cement slurry is a critical factor influencing cementing operations. If the slurry solidifies too quickly, production incidents may occur. Conversely, if the thickening process is too slow, it can cause cement slurry movement, affecting the quality of well cementing [24]. Thus, controlling the thickening time within an appropriate range is essential for meeting construction requirements.

Generally, the initial consistency of the cement slurry should be low, the thickening curve should be smooth, and the transition time should be brief. The thickening process of cement slurry with the addition of crosslinked PVA was evaluated. The basic formulation of the cement slurry is detailed as follows: (1) Jiahua G-grade oil well cement + 1% crosslinked PVA hydrogel + tap water (water-to-cement ratio of 44%); (2) Jiahua G-grade oil well cement + 1% crosslinked PVA hydrogel + 0.1% retarder G407R1 + tap water (water-to-cement ratio of 44%).

**Figure 7:**
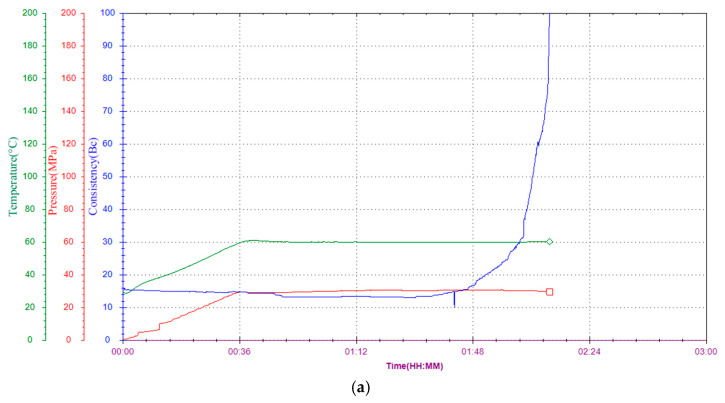

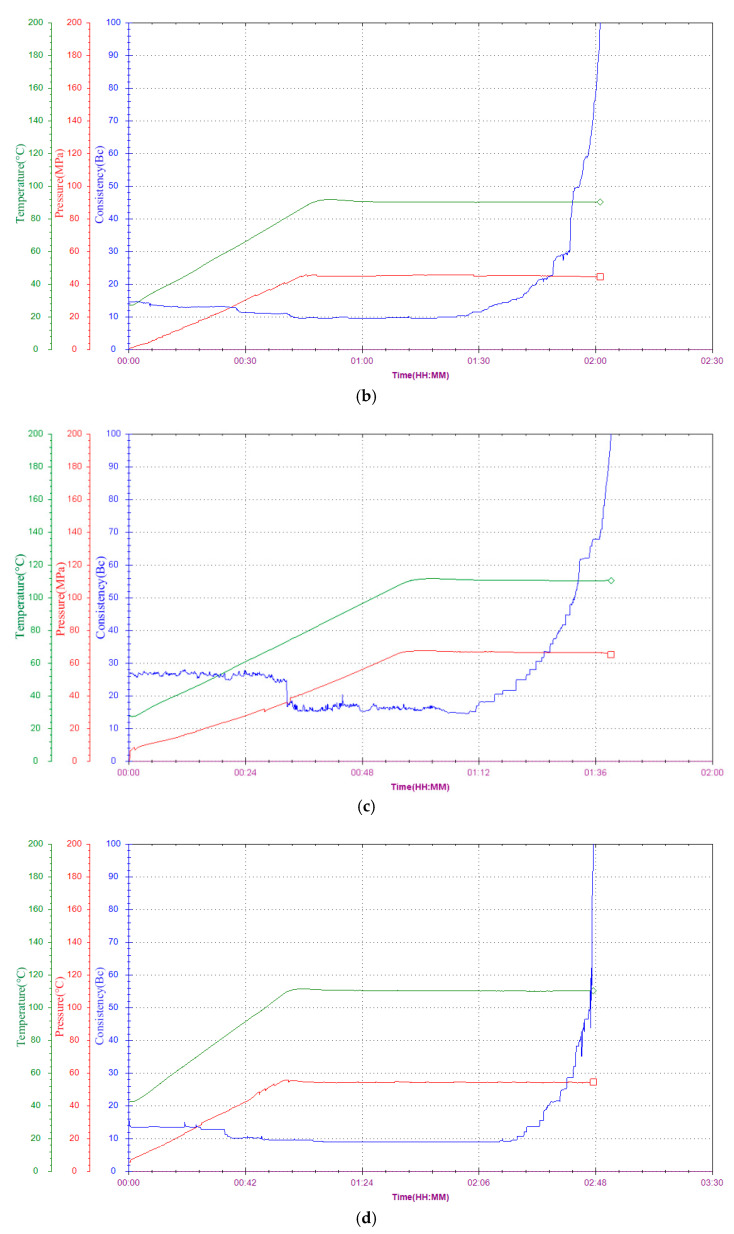
Thickening curves of cement slurry at different temperatures. ((**a**) Thickening curve at 60 °C; (**b**) thickening curve at 90 °C; (**c**) thickening curve at 110 °C; (**d**) thickening curve at 110 °C + retarding agent).

Thickening performance tests were conducted on formulation (1) at temperatures of 60 °C, 90 °C, and 110 °C, with the results shown in Figure 7a, Figure 7b, and Figure 7c, respectively.

The results indicate that as temperature increases, the thickening time decreases slightly. Initially, the thickening degree is approximately 15 Bc, and the curves exhibit a “right-angle” shape without significant “bulges”. This pattern suggests that crosslinked PVA has a moderate retarding effect, contributing to stable and predictable thickening behavior.

Figure 7d shows the thickening curve of formulation (2) which includes the retarding agent G407R1 at 110 °C. Compared to Figure 7c, this curve is smoother, with a reduced initial thickening degree and a thickening time extended by over 30 min. This result underscores the excellent compatibility between crosslinked PVA and the retarding agent, enabling improved control over the cement slurry’s thickening time.

From the above results, it is evident that at lower temperatures, the thickening time is sufficiently long to meet construction time requirements. However, at elevated temperatures, the initial viscosity increases to nearly 30 Bc, and the thickening time is shortened. This is primarily attributed to the fact that high temperatures accelerate the hydration reaction of cement, promoting the rapid formation of bridging structures between particles [25]. It is essential to incorporate retarding agents to reduce the initial viscosity and prolong the thickening time to ensure optimal performance.

### 2.8. Molecular Weight Analysis

The crosslinking reaction between PVA and glutaraldehyde results in the linkage of PVA molecular chains through crosslinking points, forming larger crosslinked units. These units exhibit significantly higher molecular weights compared to uncrosslinked PVA molecular chains. This process creates a combination of large crosslinked units and smaller uncrosslinked molecular chains, leading to a broader molecular weight distribution. Consequently, the polydispersity index (PDI) of crosslinked PVA typically increases, with the extent of this increase depending on the degree of crosslinking, as presented in Table 6.

Specifically, the average molecular weight of crosslinked PVA is 3.69 × 10^5^, and the weight-average molecular weight is 7.48 × 10^5^, both of which are significantly higher than the molecular weights prior to modification. The increase in the polydispersity index (PDI) is relatively small. Considering the crosslinking degrees of PVA and glutaraldehyde shown in Table 4 and Table 5, this increase in the PDI is reasonable.

In general, a higher molecular weight of PVA correlates with improved thermal stability, denser network structures, enhanced adhesive properties, and greater resistance to shear failure.

### 2.9. TGA Analysis

**Figure 8:**
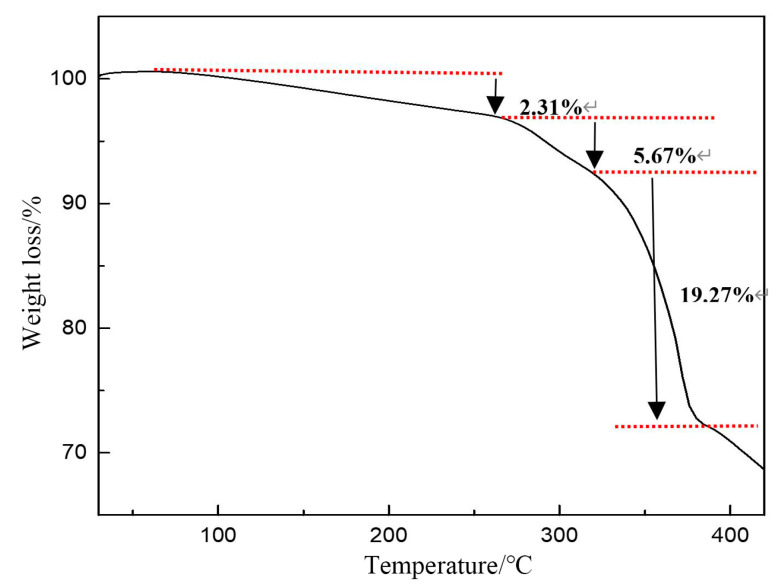
TGA of crosslinked PVA.

As the temperature increases, polymer functional groups may decompose and molecular chains may break, leading to the potential failure of polymer functionality. If crosslinked PVA undergoes thermal decomposition at a certain temperature, it can cause the cement slurry system to lose its fluid loss-reducing function, resulting in substantial fluid loss and posing significant challenges to cementing operations. Thermogravimetric analysis (TGA) can be employed to determine the temperature at which the fluid loss additive thermally decomposes, thereby determining the thermal stability and temperature limit of crosslinked PVA. Figure 8 shows that the thermogravimetric weight loss curve of crosslinked PVA is divided into three distinct stages between 28 °C and 450 °C. The first stage occurs between 30 °C and 260 °C; during this phase, the thermogravimetric rate is gradual, with a minor mass loss of 2.31%. This is attributed to the sample’s absorption of moisture, leading to dampness, and the subsequent evaporation of free or crystalline water upon heating. The second stage spans from 261 °C to 310 °C; this stage is identified as the second weight loss region, with a heat loss of 5.67%, resulting from the degradation of side chains in the crosslinked PVA. The third stage extends from 311 °C to 450 °C; in this phase, the thermal weight loss rate increases sharply, resulting in a significant mass reduction to 19.27%. This is attributed to the onset of main chain decomposition in the crosslinked PVA, which significantly impacts its properties.

The analysis indicates that crosslinked PVA remains stable up to 261 °C and can withstand high-temperature environments without undergoing thermal decomposition.

### 2.10. Zeta Potential Test

The adsorption behavior of polymeric fluid loss additives on cement particles can be evaluated using Zeta potential measurements. A lower Zeta potential value indicates greater adsorption capacity. Moreover, the Zeta potential is a critical parameter for determining the dispersibility and stability of particles, with a higher absolute value corresponding to improved stability and dispersibility [26].

**Figure 9:**
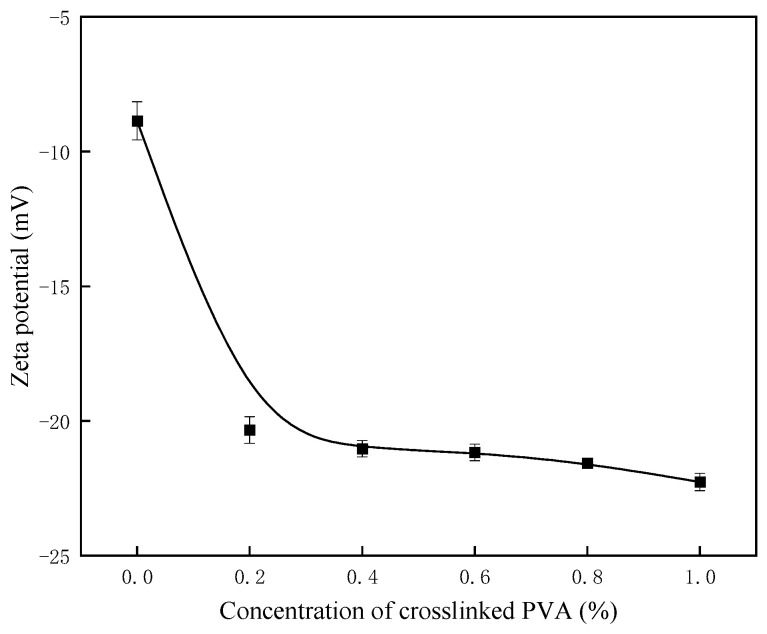
Effect of crosslinked PVA concentration on Zeta potential.

The Zeta potential values of water slurries with 0–1% crosslinked PVA were measured, and the results are presented in Figure 9. As shown, the absolute value of the Zeta potential increases progressively with the concentration of crosslinked PVA, although the rate of increase diminishes beyond a concentration of 0.2%. At a concentration of 1.0%, it reaches a maximum of −16.5 mV, indicating that crosslinked PVA exerts a strong stabilizing effect on the cement slurry.

### 2.11. Adsorption Performance Evaluation

**Figure 10:**
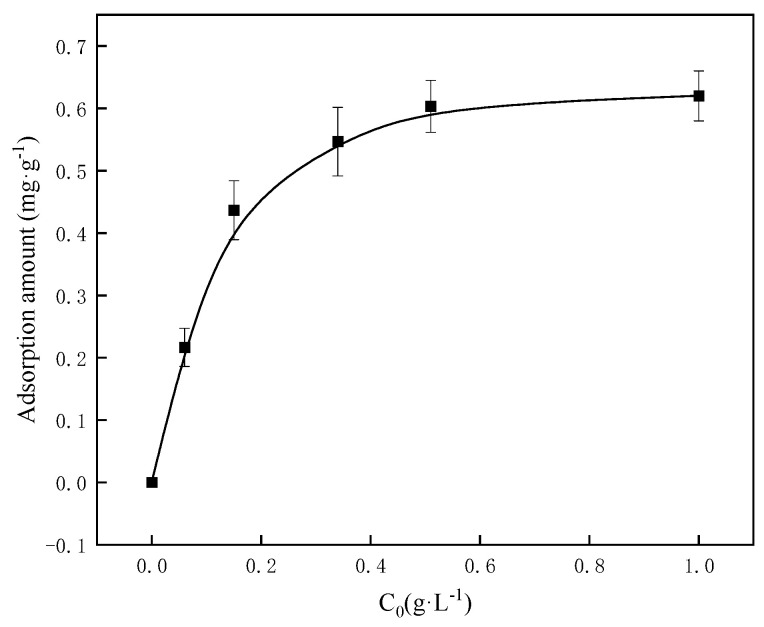
Adsorption amount of crosslinked PVA on cement particle surfaces.

The adsorption capacity of crosslinked PVA on the surfaces of cement particles was further investigated. Figure 10 reveals that the adsorption amount increases with the concentration of crosslinked PVA, stabilizing above a concentration of 0.4%, with a maximum of 0.61 mg/g with an addition of 1.0 g/L.

This observation aligns with the findings discussed in Section 2.3 and Section 2.10, demonstrating that the addition of 0.4% crosslinked PVA at low temperatures is sufficient to meet the requirements. Crosslinked PVA forms a multi-point adsorptive molecular film through hydrogen bonding between its hydroxyl groups and the silicon hydroxyl and aluminum hydroxyl groups on the surface of cement particles. Its molecular structure lacks ionic functional groups and, consequently, does not exhibit electrostatic repulsion. Consequently, the adsorption performance of crosslinked PVA is weaker than that of polymers containing ionic functional groups [27].

### 2.12. Scanning Electron Microscopy (SEM)

**Figure 11:**
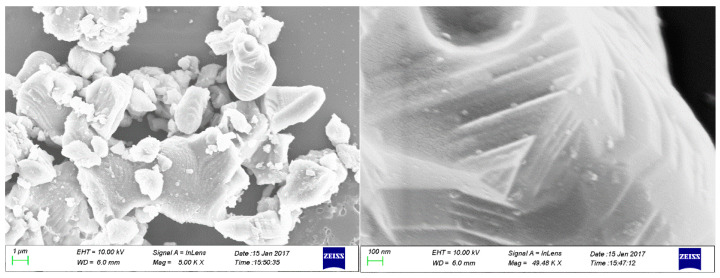
SEM image of cement slurry after solidification.

The cross-sectional morphology of hardened cement slurry was examined using scanning electron microscopy (SEM), as shown in Figure 11. The SEM images depict cement stone particles stacked and bonded together, forming a stable structure. This structure is hypothesized to result from the interaction between crosslinked PVA and cement particles, where cement particles accumulate and adhere to one another through the mediation of crosslinked PVA molecules. These interactions are believed to lead to the formation of a thin molecular film that enhances the overall density of the cement slurry structure. The adsorption of crosslinked PVA onto cement particle surfaces, along with the sealing of inter-particle pores, synergistically contributes to the reduction in fluid loss.

### 2.13. Mechanism

In oil and gas field cementing operations, during the process of cement slurry circulation or while waiting for the cement to set in the annulus, a pressure differential between the liquid column and the formation can result in the migration of free water from the cement slurry to the formation. This phenomenon, referred to as cement slurry dehydration [28], leads to water loss and the subsequent formation of a filter cake. As fluid loss occurs through this filter cake, reducing its permeability becomes critical for controlling fluid loss.

**Figure 12:**
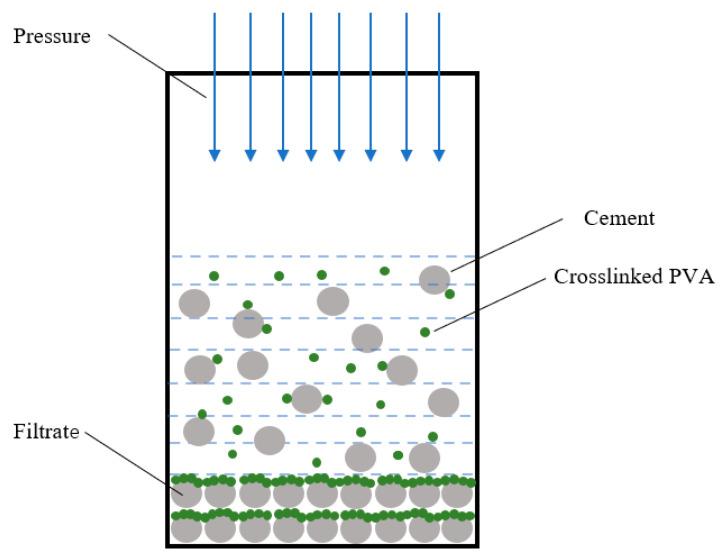
Water loss mechanism of cement slurry and water loss reduction mechanism of crosslinked PVA.

Research findings indicate that the adsorption of crosslinked PVA on the surface of cement particles and the blocking effect of the strong hydrogel are the primary mechanisms responsible for mitigating fluid loss. As illustrated in Figure 12, during the filtration process, cement particles and the polymer gel within the cement slurry are driven toward the filter screen due to the pressure differential. Near the filter cake and screen, the concentration of cement particles and the polymer increases, enhancing the hydrogel’s viscoelasticity and its capacity to block pores effectively.

Additionally, when cement particles interact with polymer molecules, the polymer molecules act as binders while the cement particles serve as bridges for mutual adhesion, forming a continuous gel layer. During the initial stages of dehydration (typically within a few seconds), prior to polymer film formation, significant instantaneous dehydration occurs, exhibiting a “threshold effect” of dehydration [29]. Once the polymer gel film develops, continued fluid loss causes the film to thicken, reducing the pressure differential across it.

Furthermore, the polymer molecules within the accumulated filter cake create a discontinuous gel structure that further minimizes water loss. The crosslinked PVA utilized in this study forms a network-structured hydrogel with high strength in aqueous environments due to its chemically bonded structure. The resulting adsorption film is uniform, dense, strong, and thermally stable, significantly enhancing both the fluid loss control and temperature resistance of the PVA hydrogel.

## 3. Conclusions

This study optimized the synthesis process of crosslinked PVA and incorporated the crosslinked PVA into cement slurry as a fluid loss additive, evaluating its effectiveness, structural characteristics, and mechanism of action. The optimal conditions for the crosslinking reaction of PVA were determined as follows: a reaction temperature of 50 °C, a polyvinyl alcohol mass concentration of 8%, a glutaraldehyde-to-PVA hydroxyl group molar ratio of 1.47, and an acid concentration of 1 mol/L.

The filtration, rheological properties, stability, and thickening performance of the crosslinked PVA in oil field cement slurry were evaluated. The results indicate that the fluid loss additive comprising the crosslinked PVA could be maintained below 50 mL under test conditions. The temperature resistance could be increased to at least 110 °C. The rheological properties, free liquid, and thickening performance can meet construction requirements.

TGA demonstrated that crosslinked PVA undergoes significant thermal decomposition above 261 °C, indicating excellent thermal stability. SEM, Zeta potential, and adsorption performance analyses revealed that the adsorption of crosslinked PVA on cement particles and the blocking effect of the high-strength hydrogel are the primary mechanisms behind reducing fluid loss. This study provides a highly efficient fluid loss additive for oil field cementing applications and offers valuable insights for the development of similar materials in related studies.

## 4. Materials and Methods

### 4.1. Materials and Reagents

PVA-1788, glutaraldehyde, and other (AR) solvents were obtained from Tianjin Kemio Chemical Reagent Co., Ltd. Table 7 outlines the degrees of hydrolysis (DHs) and polymerization (DP) for polyvinyl alcohol (Tianjin, China). Cement was sourced from Jiahua Cement Plant (Nanchong, China). Table 8 and Table 9 present the chemical composition and physical properties of the cement, respectively. The dispersant G408FCJ and retarding agent G407R1 were provided by CNPC Chuanqing Drilling Engineering Co., Ltd. (Xi´an, China).

### 4.2. Synthesis of Crosslinked PVA

**Figure 13:**
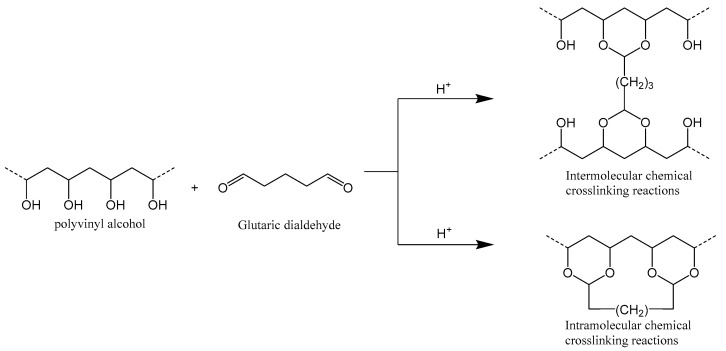
Crosslinking reaction of PVA and glutaraldehyde.

Glutaraldehyde was employed as a crosslinking agent to synthesize a crosslinked PVA hydrogel with a three-dimensional network structure through chemical crosslinking with PVA (Figure 13). By optimizing the ratio of glutaraldehyde to PVA and refining the reaction conditions, the degree of crosslinking and the hydrogel’s fluid loss additive properties were significantly improved. This optimization enhances its fluid loss-reducing capabilities, rendering it more suitable for application as a fluid loss additive in oil wells.

We weighed a specific amount of PVA and distilled water, stirring the mixture with an electric mixer. We heated the mixture to 50 °C and stirred it for about 20 min to ensure complete dissolution. We cooled the mixture to the desired experimental temperature, then added glutaraldehyde and stirred for an additional 40 min. We adjusted the pH to an acidic range by adding hydrochloric acid, followed by neutralization using a sodium hydroxide solution. We cooled the mixture to room temperature to obtain the crosslinked PVA hydrogel, which was characterized and evaluated for its performance.

### 4.3. Performance Assessment

Performance tests on the cement slurry were conducted in accordance with the Chinese Petroleum and Natural Gas Industry Standard SY/T 5546-92 “Test Method for Application Performance of Oil Well Cement” [30].

### 4.4. Thermogravimetric Analysis

Thermogravimetric analysis (TGA) of the synthesized crosslinked PVA was conducted using the DSC823 TGA/SDTA85/e thermal analyzer from Mettler Toledo (Greifensee, Switzerland). The analysis was performed in a nitrogen atmosphere over a temperature range of 40 °C to 600 °C, with a heating rate of 10 °C/min.

### 4.5. Molecular Weight Analysis

The molecular weights of PVA and crosslinked PVA were measured by dissolving the polymers in tetrahydrofuran as the mobile phase, with a flow rate of 1 mL/min. The temperature was maintained at 30 °C, and polystyrene (PS) was used as the standard. The molecular weight measurement range was from 500 to 3,000,000.

### 4.6. Zeta Potential Measurement

Zeta potential measurements of cement particles were performed immediately after mixing, following API specifications, using the Brookhaven NanoBrook Omni multi-angle particle size and high-sensitivity Zeta potential analyzer (Alkmaar, Netherlands). A water-to-cement ratio of 0.44 was maintained throughout the measurements.

### 4.7. Adsorption Performance Evaluation

The adsorption performance of crosslinked PVA on cement particles was evaluated by mixing Jiahua G-grade cement with varying concentrations of crosslinked PVA solution. The mixture was thickened at a certain temperature, then centrifuged at 4000 r/min for 15 min, and the supernatant was analyzed using the potassium dichromate oxidation method to determine the concentration of crosslinked PVA in the filtrate. This enabled the calculation of the amount of crosslinked PVA adsorbed onto cement particles.

## Figures and Tables

**Table 1:** Levels of factors in uniform experimental design.

Factors	Level						
	1	2	3	4	5	6	7
A	30	40	50	60	70	80	90
B	12	10	8	6	4	2	1
C	1.22	1.34	1.42	1.54	1.59	1.65	1.69

**Table 2:** Uniform experimental design and experimental results at various levels. Values are mean + standard deviation of three replicates.

Number	Reaction Conditions			Degree of Crosslinking (%)
	Factor A	Factor B	Factor C	
1	30	12	1.22	1.60
2	40	10	1.59	2.08
3	50	8	1.65	1.79
4	60	6	1.69	1.49
5	70	4	1.54	1.05
6	80	2	1.34	0.78
7	90	1	1.42	1.04

**Table 3:** The influence of crosslinked PVA cement slurry on rheological properties expressed as the mean ± standard deviation of three replicates.

Concentration (%)	Temperature (°C)	n	K
0	50	0.4470	5.5055
0.4	50	0.8746	0.3111
0.6	50	0.8063	0.6494
0.8	50	0.8075	0.9925
0	80	0.3860	4.7863
0.4	80	0.7668	0.4324
0.6	80	0.8325	0.4151
0.8	80	0.8217	0.6385
0	110	0.7859	0.0722
0.4	110	0.8146	0.2911
0.6	110	0.9190	0.2592
0.8	110	0.8978	0.2592

**Table 4:** Effects of crosslinked PVA on compressive strength of cement slurry. Values represent mean ± standard deviation of three replicates.

Temperature (°C)	Concentration (%)	Compressive Strength (MPa/24 h)
50	0	24.5
50	1	26.6
70	0	27.2
70	1	28.5
90	0	29.5
90	1	29.3
110	0	32.7
110	1	32.2

**Table 5:** Effect of crosslinked PVA on free water in cement slurry. Values represent mean ± standard deviation of three replicates.

Temperature (°C)	Concentration (%)	Free Water Content (%)
50	0	2.7
	0.5	0.9
	1.0	0.6
	1.5	0
70	0	2.5
	0.5	1.0
	1.0	0.6
	1.5	0
90	0	2.2
	0.5	0.5
	1.0	0
	1.5	0
110	0	1.9
	0.5	0.5
	1.0	0
	1.5	0

**Table 6:** Molecular weight test results of crosslinked PVA.

Polymer	M_n_	M_w_	PDI = M_w_/M_n_
PVA	1.75 × 105	3.01 × 105	1.72
Crosslinked PVA	3.69 × 105	7.48 × 105	2.03

**Table 7:** Degrees of hydrolysis (DHs) and degrees of polymerization (DPs) of PVA used.

PVA	Degrees of Hydrolysis (DPs)	Degrees of Polymerization (DHs)
1788	1700	99%

**Table 8:** Chemical composition of cement (%).

CaO	SiO_2_	Fe_2_0_3_	Al_2_O_3_	MgO	K_2_O	Na_2_O	Loss	Other
62.25	20.46	4.27	3.81	2.54	0.36	0.30	2.14	3.87

**Table 9:** Physical and mechanical properties of Jiahua G-grade cement.

Initial Setting Time (min)	Final Setting Time (min)	Specific Surface Area (m^2^/kg)	Flexural Strength (MPa)		Compressive Strength (MMPa)	
180	250	345	3 d	28 d	3 d	28 d
			6.1	7.9	31.6	53.2

## Data Availability

The data presented in this study are openly available in article.
